# Supplementary material for: Remdesivir and dexamethasone as tools to relieve hospital care systems stressed by COVID-19: A modelling study on bed resources and budget impact
Source: PLoS One. 2022 Jan 12;17(1):e0262462. doi: 10.1371/journal.pone.0262462 (PMC8754316; doi:10.1371/journal.pone.0262462)
Supplement: Attachment — Submitted filename: review manuscript.docx [file pone.0262462.s002.docx]

I recommend this study for publication; however, I would ask you to consider the following points:

1) Please include the introduction epidemiological data from the context of the COVID-19 in France.
2) Please include a table with the probabilities of hospital admission, admission to the ICU and mortality adjusted for sex and comorbidities indicated in the methodology since it is important for the development of the model carried out.
3) In the subgroup of patients receiving high-flow oxygen or non-invasive ventilation (subgroup 6 of study ACTT-1) no statistically significant differences were observed in the length of hospital stay (Rate ratio: 0.82 (0.40–1.69) since 1 is within the confidence interval. Please, I propose to the authors to be conservative and not estimate this reduction in modeling.
4) Spend the next sentence of the methodology to the discussion or delete it: “This proportion was similar to the 35.2% that was seen in 150 a prospective cohort study that included 4,643 patients from 138 hospitals in France, Belgium 151 and Switzerland between February and May 2020 (21).”
5) Please discuss the results with other simulations carried out in other countries or other studies.
